# Applications of unmanned aerial vehicles in Antarctic environmental research

**DOI:** 10.1038/s41598-021-01228-z

**Published:** 2021-11-05

**Authors:** Antonio Tovar-Sánchez, Alejandro Román, David Roque-Atienza, Gabriel Navarro

**Affiliations:** Institute of Marine Sciences of Andalusia, ICMAN (CSIC), Campus Río San Pedro, 11510 Puerto Real, Cádiz Spain

**Keywords:** Environmental sciences, Ecology, Biodiversity, Community ecology

## Abstract

Antarctica plays a fundamental role in the Earth's climate, oceanic circulation and global ecosystem. It is a priority and a scientific challenge to understand its functioning and responses under different scenarios of global warming. However, extreme environmental conditions, seasonality and isolation hampers the efforts to achieve a comprehensive understanding of the physical, biological, chemical and geological processes taking place in Antarctica. Here we present unmanned aerial vehicles (UAVs) as feasible, rapid and accurate tools for environmental and wildlife research in Antarctica. UAV surveys were carried out on Deception Island (South Shetland Islands) using visible, multispectral and thermal sensors, and a water sampling device to develop precise thematic ecological maps, detect anomalous thermal zones, identify and census wildlife, build 3D images of geometrically complex geological formations, and sample dissolved chemicals (< 0.22 µm) waters from inaccessible or protected areas.

## Introduction

Climate change is rapidly affecting polar regions in different ways (e.g. change in their productivity, biodiversity, ecosystems and their functioning in general) with consequences that extend to the whole planet^1^. It is therefore a priority, and a scientific challenge, to understand the functioning of the polar ecosystems in order to predict and reduce risks at both regional and global scales. Antarctica, including the continent and surrounding Southern Ocean, remains one of Earth’s under-explored regions with very sparse data coverage, which hampers the efforts to understand its ecological functioning, and its responses under different scenarios of global warming. Its harsh environment, remoteness, inaccessibility, and logistical constraints render any environmental study a challenge for the scientific community. This challenge becomes more complicated with the need for a multidisciplinary approach in environmental studies, which generally requires that the collection of samples and data be interpreted from a chemical, physical, geological and/or biological perspective.

Deception Island, with more than twenty identified eruptions over the past two centuries, is the most active volcano in the South Shetland Islands^2^. Because of its unique environment with exceptional, diverse and particular flora, the Antarctic Treaty gives legal protection to 11 parts of the island, being designated as an Antarctic Specially Protected Area (ASPA)^3^. The island has, among many of its values: (i) the greatest number of rare (i.e., known to grow only in few places in the Antarctic and often in small quantity) and extremely rare (i.e., known to grow in only one or two places in the Antarctic) plant species (mosses and lichens) of any site in the Antarctic; (ii) different bryophyte communities developed under microclimate produced by geothermal activity; (iii) a unique community of brackish-water algae present in the only intertidal lagoon with hot springs in Antarctica (Kroner Lake). In addition to its outstanding environmental values it is also protected for its scientific values (i.e., for terrestrial biology, zoology, geomorphology and geology). According to the Antarctic Treaty, the Management of the Area aims to prevent unnecessary human disturbance and avoid scientific research that endangers the natural ecological system or requires excessive sampling of the flora^3^. Currently, only the following two research stations operate on the island during the austral summer: “Gabriel de Castilla” (Spain) since 1989 and “Decepción” (Argentina) since 1948. Multiple research studies have been carried out on the island and have contributed notably to the knowledge of the functioning of the island in different scientific disciplines and with different targets, such as pollution^4,5^, biodiversity^6,7^, volcanology and geothermal^8^, geochemistry^9^, among others. Nonetheless, multidisciplinary studies that gather information from multiple specific areas of expertise remain scarce. Antarctic research is usually constrained by: (i) short operational time, usually limited to the austral summer when most countries operate from their Antarctic bases; (ii) human resources, which are also limited by the capacity of the research stations; (iii) inaccessibility or difficulty in accessing the study areas; (iv) harsh environment coupled with changing, adverse and unpredictable weather; and (v) the need to carry out studies while avoiding any impact on the existing flora and fauna or that imply environment modification. Therefore, Antarctic environmental studies require the use of technological tools that minimize these issues, collect the maximum number of data and samples in order to facilitate multidisciplinary research, and maximize and improve the interpretation of information obtained in the field.

The use of UAV technology as a remote sensing platform has brought about a revolution in environmental monitoring. Compared with conventional remote sensing techniques, such as satellites imagery, UAVs can provide data with higher temporal and spatial resolution, and are not limited by cloud cover. UAVs significantly reduce the cost of the research since they require less infrastructure and personnel to access the study area. Although UAVs may disturb wildlife during takeoff or flying at low altitudes^10^, they are less invasive than human on foot^11^, and reduce human risk during data and sampling collection on the field, particularly in areas of difficult access. Here we present images obtained with different sensors onboard UAVs for environmental studies on Deception Island. The survey aims to highlight the potential of UAV technology to collect surface water samples, multispectral and thermal images and data with high spatial and temporal resolution, minimal operational time-consumption, low risk for scientists and minimal impact on the natural environment.

## Results and discussion

### Identification and characterization of biotic and abiotic components in a penguin colony using RGB and multispectral cameras

**Figure 1:**
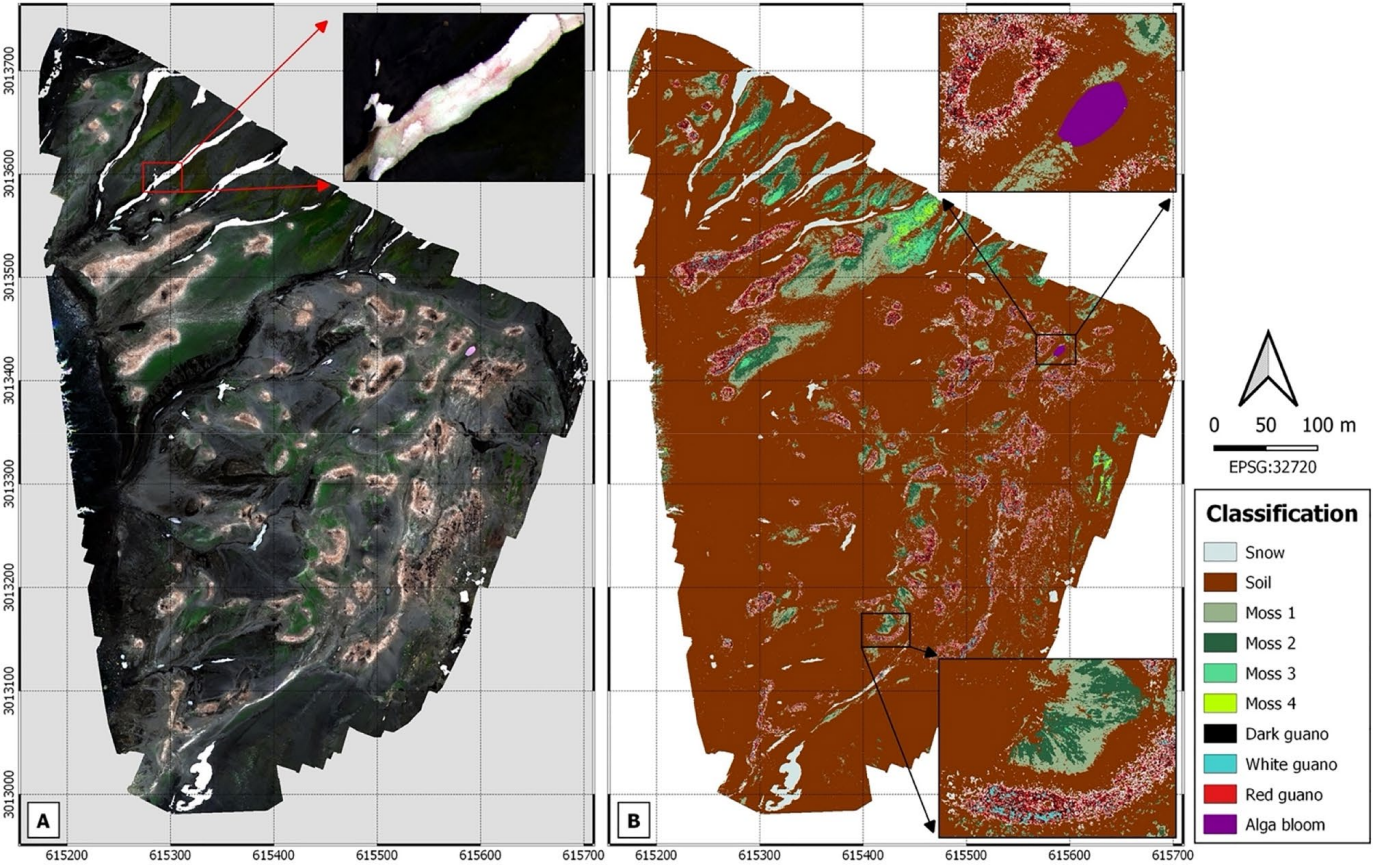
Photomosaics of Vapour Col Chinstrap penguin colony on Deception Island composed of 3800 pictures taken at 100 m altitude with a 10 bands multispectral camera onboard a hexacopter, achieving 6 cm/pixel size. Panel (**A**): visible RGB mosaic (Red-668, Green-560 and Blue-475) with a zoom capture showing red snow patch; Panel (**B**): thematic map generated through non-supervised classification method.

Figure 1 shows two image mosaics of a Chinstrap penguin (*Pygoscelis antarcticus*) colony, composed of 3800 pictures taken during a 29-min flight at 100 m altitude with a multispectral camera (MicaSense RedEdge-MX) using RGB bands (i.e., Red-668, Green-560 and Blue-475) (Fig. 1A) and the 10 wavelength bands covering the spectrum from visible to near-infrared light (Fig. 1B). With a resolution of 6 cm/pixel, penguin nests are clearly visible in the RGB mosaic, which are characterized by the absence of vegetation and with a predominant pink/brown color due to the abundance of guano deposition. The RGB mosaic also shows snow patches (white color), moss beds (green color) and one small lagoon with a bloom of red-pigmented greenalgae (*Chlorophyceae*) (Fig. 1A, upper right corner). Red algae (*Chlamydomonas nivalis*) patches on snow and ice are visible by zooming into a region of ice (Fig. 1A). More detailed information is obtained when the light spectrum from visible to near-infrared is used. Using the 10 wavelength bands, a thematic map was generated with the QGIS software and using a non-supervised classification method (Fig. 1B). Here it is possible to distinguish up to four species of mosses and three types of penguin guano that was verified with field observations.

Deception Island harbors up to 54 species of mosses, of which 13 species (including two endemics) have not been recorded elsewhere in the Antarctic. This, together with eight species of liverwort and 75 species of lichen, makes Deception Island an exceptional and unique place in Antarctica with legal protection under the Antarctic Treaty^3^. The use of a multispectral sensor onboard the UAV provides unique information to detect, classify and monitor moss beds without anthropogenic impacts. Antarctic moss bed health has already been assessed using multispectral sensors onboard UAVs^12^. Taxonomic identification would be feasible by indentifying previously each species in the field and later assigning the spectral signature using the UAV, as recently suggested by Miranda et al. (2020), who monitored lichens and mosses in the Antarctic using a combination of satellite imagery and UAVs^13^.

Penguin guano has been suggested to be an important source of bioactive metals (e.g. Cu, Fe, Mn, Zn) for the sea surface waters, potentially fueling primary production of the Southern Ocean^14^. It has been suggested that the penguin species that feed mainly on Antarctic krill (*Euphausia superba*) (i.e., Chinstrap: *Pygoscelis antarcticus*, Adélie: *Pygoscelis adeliae* and Gentoo: *Pygoscelis papua*) excrete the highest concentrations of these bioactive metals^15^. Guano from these three congeneric penguin species has revealed the presence of microplastics across the Antarctic^5^. However, in order to estimate the magnitude of penguin fecal products that reach the sea, it is necessary to quantify the amount of guano excreted by the penguin colonies on land. This is possible with the multispectral reflectance data obtained from the UAV, which not only identify the guano coverage but also distinguishes different types of guano. Guano color is the result of diet, which, in turn, is related to the phase of the breeding cycle; therefore, a diet rich in krill is characterized by an excretion of pink guano, while a diet predominantly based on fish implies white guano^16^. Dark guano is the result of the mixture of guano with the soils that produce mud during wet precipitation.

It is increasingly common in the Arctic and Antarctic to find well-developed algae blooms as highly visible red patches on the snow surface caused by red-pigmented green algae (*Chlorophyceae*), and that produce the phenomenon commonly-known as red snow^17^. These algal blooms play a crucial role in decreasing the snow-surface albedo and, consequently, accelerating the melt rate, as well as in nutrient and carbon cycling^18,19^. Mapping and monitoring the extent of snow algal blooms have so far been focused on satellite remote sensing; however, the spectral, temporal and spatial resolution of multi-spectral satellite imagery limits the study of most snow and ice algae^18^. Images taken from our UAV can enable the detection of patches of red snow on the surface snow with centimetric resolution (Fig. 1A). In addition, the image mosaic reveals the existence of a red snow bloom in a small pond located in a valley inside the colony (Supplementary Fig. S1). To the best of our knowledge, the existence of this bloom has not been previously reported and its monitoring could provide relevant information about the formation and proliferation of this bloom and its impact on cryospheric environments.

As a whole, the image mosaic of the Chinstrap penguin colony in Vapour Col (the second largest breeding colony in the island with about 12,000 pairs of penguins^20^) may provide unique information about the different ecological niches linked to a penguin colony and their interactions. For example, the distribution and type of guano as nutrient and metal sources could be influencing the distribution and speciation of the flora in the area.

### 3D geological formation using RGB camera

**Figure 2:**
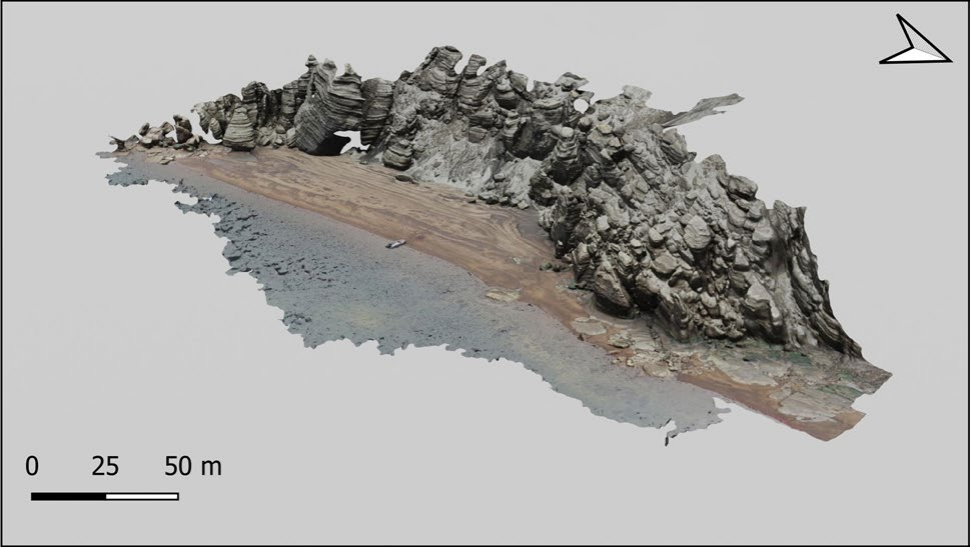
3D photogrammetry of the Murature formation built with 843 RGB pictures taken from the RGB Hasselblad camera quadcopter DJI Mavic 2 Zoom at 40-m altitude, achieving 1.4 cm/pixel size.

Deception Island is a complex volcanic system formed as a result of the explosive eruption of basaltic-to-andesitic magmas^21^. Among its multiple structures and stratigraphy, we surveyed the Murature formation, a consolidated andesitic lapilli tuff^22^. Using the quadcopter with a RGB camera and the software Pix4D we created a 3D photogrammetry of the Murature formation (Fig. 2; Supplementary Movie S1). The software uses a Structure from Motion photogrammetry algorithm, where obtained 3D points are interpolated to form a triangulated irregular network in order to obtain digital Surface model (DSM). This DSM is then used to project every image pixel and to calculate the georeferenced orthomosaic. For the Murature formation, the photogrammetry was generated with 843 pictures obtained from three 20-min flights at an altitude of 40 meters, taking pictures from two different angles to obtain the heights of the features (60° and 90°). With 1.4 cm/pixel resolution the resulting mosaic provides a unique view of the geological formation that will support the study of how the rocks were formed and its evolution in relation to the various geological processes that occurred on the island. 3D photogrammetry is also useful in geomorphological research. Specifically, in Deception Island morphometrics studies of landform (e.g. Crater and cone diameters, depths, slopes, heights, etc.) are useful to estimate the eruptive recurrence of the island, and in turn, for advising volcanic hazards^23^.

### Thermal imagery to estimate animal abundance and to detect thermal anomalies

**Figure 3:**
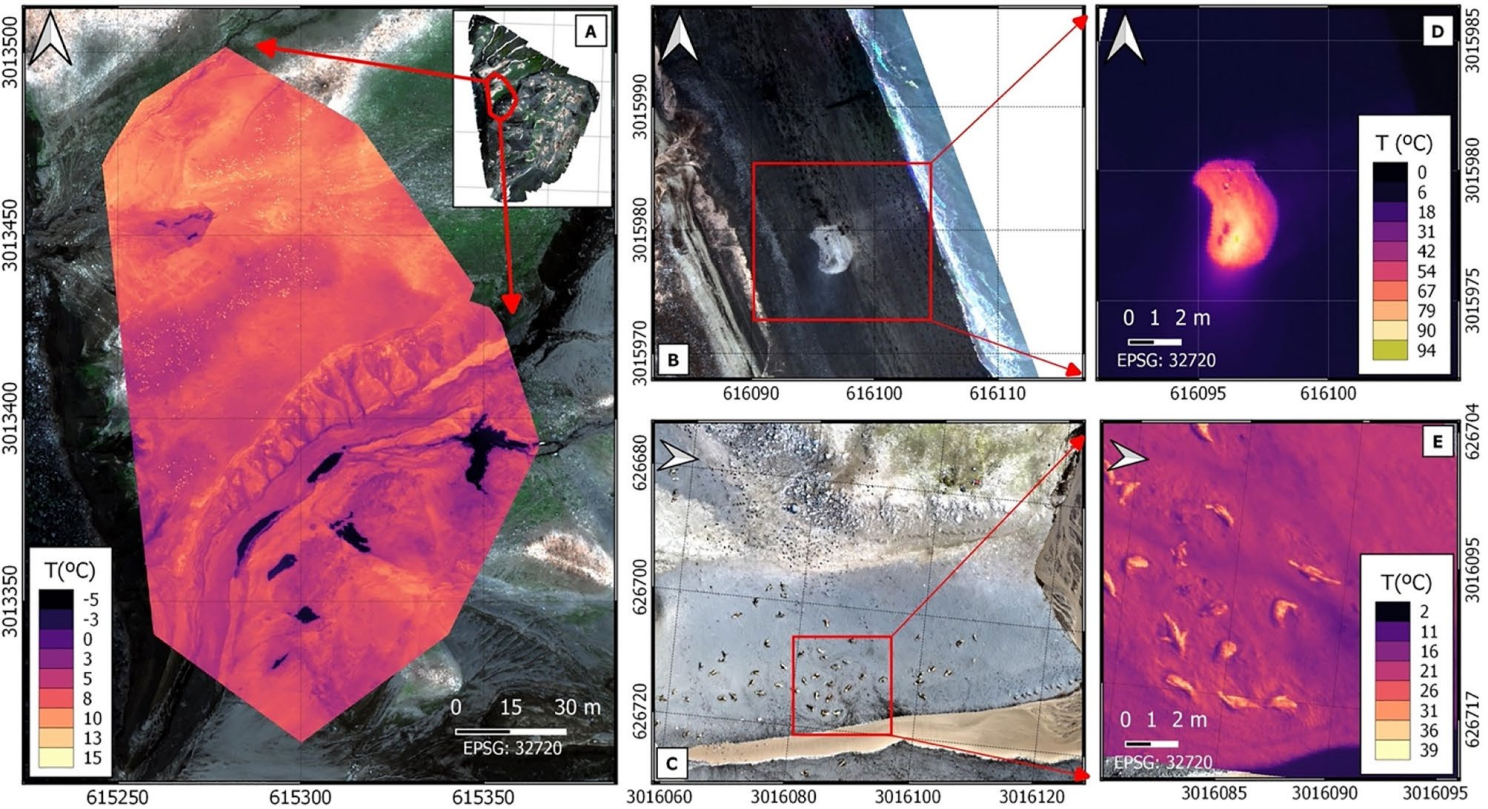
Thermal imagery. Panel (**A**): thermal mosaic of a section of Vapour Col (8.5 cm/pixel). Penguins are distinguished throughout the colony as small dots around 15 °C; Panel (**B**) and (**D**): RGB (Red-668, Green-560 and Blue-475 bands) and thermal picture of fumarole at Fumarole Bay (5.4 cm/pixel), respectively; Panel (**C**) and (**E**): RGB and Thermal image of Fur seals at Baily Head (5.4 cm/pixel), respectively.

The combination of UAV technology with a thermal-imaging camera is very useful for studying and monitoring wildlife and thermal anomalies on Deception Island. Chinstrap penguin and fur seal (*Arctocephalus gazella*) heat signatures were detected at Vapour Col and Baily Head, respectively (Fig. 3A, E). Figure 3A shows a mosaic from a Vapour Col section composed of 336 images taken with a thermal camera (FLIR Vue Pro R) onboard the hexacopter during a 29-min flight at 100 m altitude, whereas Fig. 3C shows one thermal picture of fur seals at Baily Head. Penguins and fur seals, with a thermal signature of 15 °C and 26 °C, respectively, are clearly identified. Penguins are highly sensitive to climate change and are considered “marine sentinels” for quantifying environmental change in the Southern Ocean^24^. However, the distribution and population dynamics of species such as the Chinstrap penguin are not well understood, mainly because they nest in remote and rugged areas, on-the-ground census work is difficult and sporadic^25^. As demonstrated for Adelia penguins^26^ the use of thermal imagery would allow reliable population estimates of Chinstrap penguins. Even, the use of RGB aerial images for animal counting would be far more accurate than from land-based surveys. Nevertheless, the scientific challenge is to develop a machine learning algorithm that can distinguish between animal species, based on their morphology and unique thermal fingerprint, which is only feasible using the high resolution provided by UAVs.

Other useful application of thermal cameras onboard UAVs on Deception Island is the easy and precise detection and monitoring of thermal anomalies. Figure 3B–D shows a thermal picture of one of the multiple fumaroles on the island, reaching temperatures above 90 °C. Seismic monitoring of volcanos on Deception Island has being ongoing since 1986, including many recorded volcano-tectonic earthquakes, long-period events and volcanic tremor^27^. There have been six documented volcanic eruptions on the island between 1841 and 1971^28^, nowadays volcanic and geothermal activities are limited to fumaroles and hot sands. Monitoring of these fumaroles using UAVs can provide a key in surveillance for early warming systems alerting of volcano activity on the island. UAVs not only accurately detect changes in temperature but also allow the increase in monitoring frequency when required.

### Surface water sampling

**Figure 4:**
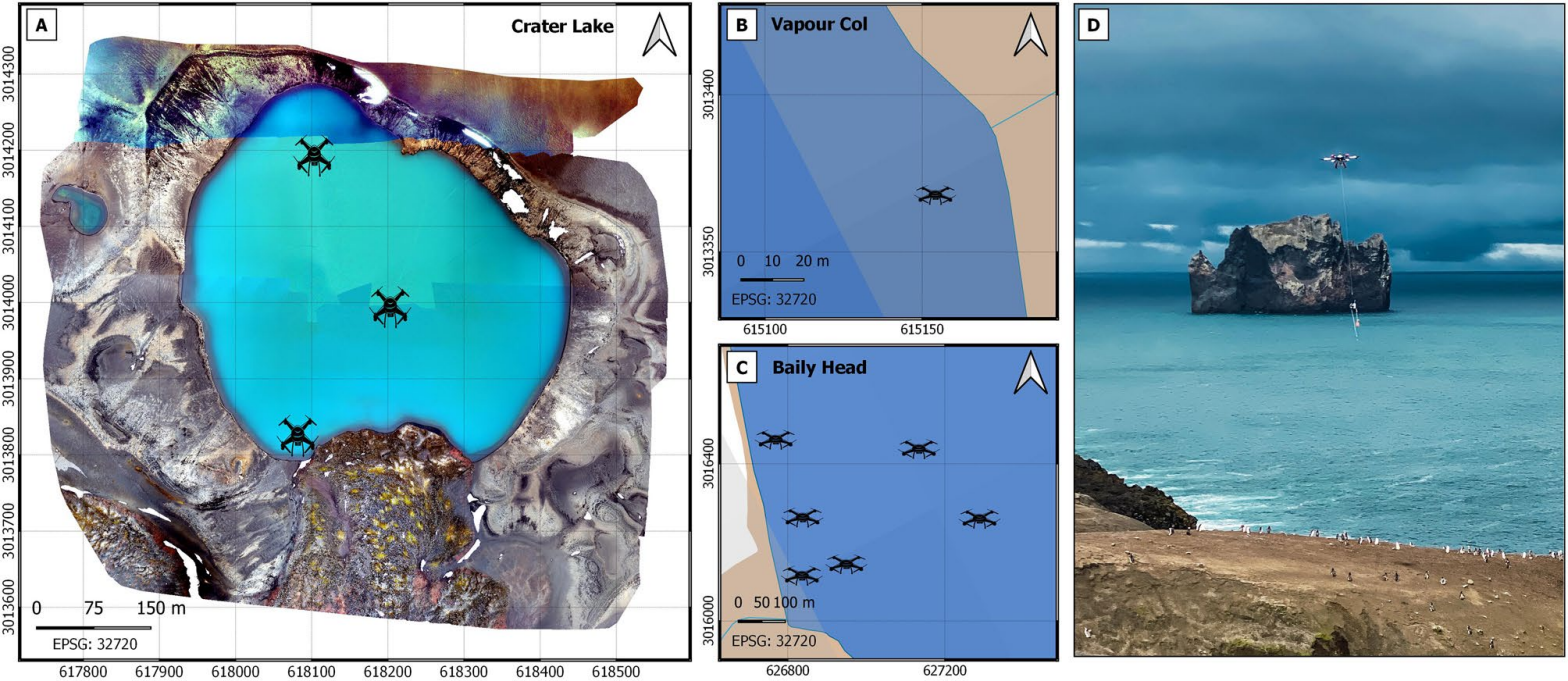
Locations of surface water samples collected in Crater lake (**A**), Vapour Col (**B**), and Baily Head (**C**) using aerial water sampling device, and picture of the UAV (hexacopter) carrying, at 100 m altitude, the water sampling device and the multiparametric instrument (**D**). Stations at Crater lake are plotted on a mosaic composed of 3096 pictures taken during three flights of 14 min each at 120 m altitude using a quadcopter with an integrated RGB camera and a multispectral camera array with 5 bands, achieving 6.5 cm/pixel size.

UAVs provide unique opportunities for remote sample collection from surface waters, particularly in harsh or dangerous environments. Using a surface water sampling device described in the sampling and method sections we collected filtered fresh and saline surface waters at: (1) Three locations in Crater Lake (Fig. 4A). Crater Lake is part of the Antarctic Specially Protected Area (ASPA 140) due to its exceptional botanic and ecological value^3^. The use of drones for water sampling avoids human disturbance through the transportation and use of infrastructure, such as inflatable boats, and the risk that they pose to the natural ecological system. (2) One and six coastal locations in the Vapour Col and Baily Head penguin colonies, respectively (Fig. 4B, C). Access to the coastal zone inhabited by penguins requires approaches by boat (often assisted by an oceanographic vessel). The approaches do not only disturb the penguins that enter and exit the colony but, due to the coastal orography and waves, also dangerously hinders such an operation. The surface water sampling device onboard the UAV allowed in-situ water collection, minimizing the risk of impact on flora and fauna, limiting water disturbance and preventing contamination in the trace metal analysis. Attached to the sampling system we included a small multiparametric instrument referenced with time and GPS position to measure ancillary parameters, such as conductivity, temperature and depth (CastAway-CTD^®^) (Fig. 4D). The aerial water sampling has been validated for trace metal analysis using ICP-MS by comparing metal concentrations of samples collected in a saline pond with the surface water sampling device onboard the UAV (i.e. average ± SD, n = 3; Ti: 0.20 ± 0.09; V: 1.92 ± 0.07; Cr: 1.5 ± 0.1; Mn: 19.4 ± 0.4; Fe: 11.6 ± 0.5; Cu: 1.9 ± 0.2; Zn: 0.5 ± 0.3; all values in ppb) and the traditional peristaltic pump system used from land or on boats^29^ (i.e. average ± SD, n = 3; Ti: 0.20 ± 0.06; V: 1.93 ± 0.09; Cr: 1.3 ± 0.1; Mn: 19.1 ± 0.3; Fe: 11.8 ± 0.3; Cu: 2.1 ± 0.4; Zn: 0.4 ± 0.3; all values in ppb).

Deception Island is an example of the complexity of Antarctic environments, where environmental research studies need to deal with the inter- and multi-disciplinary analysis of processes, such as volcanic and geothermal activities, limnological process from its multiple lakes and ponds, sparse and exceptional flora and diverse fauna, among other. UAV surveys on Deception Island have demonstrated that this technology may substantially contribute to the progress in environmental biological, geological and chemical studies. UAVs permit researchers to study environmental processes at smaller spatial and temporal scales compared to other remote platforms (e.g. satellites), in a more cost-effective and safer way than on foot studies. Furthermore, they are less invasive and less disturbing to wildlife and the ecosystem. The simultaneous use of multi-sensors for multiple applications and the development of algorithms based on images obtained from the drone to detect, classify and count animals in real time are the new challenges that would significantly contribute to the study of the functioning of the Antarctic ecosystem and its ongoing environmental processes.

## Methods

### Study area

**Figure 5:**
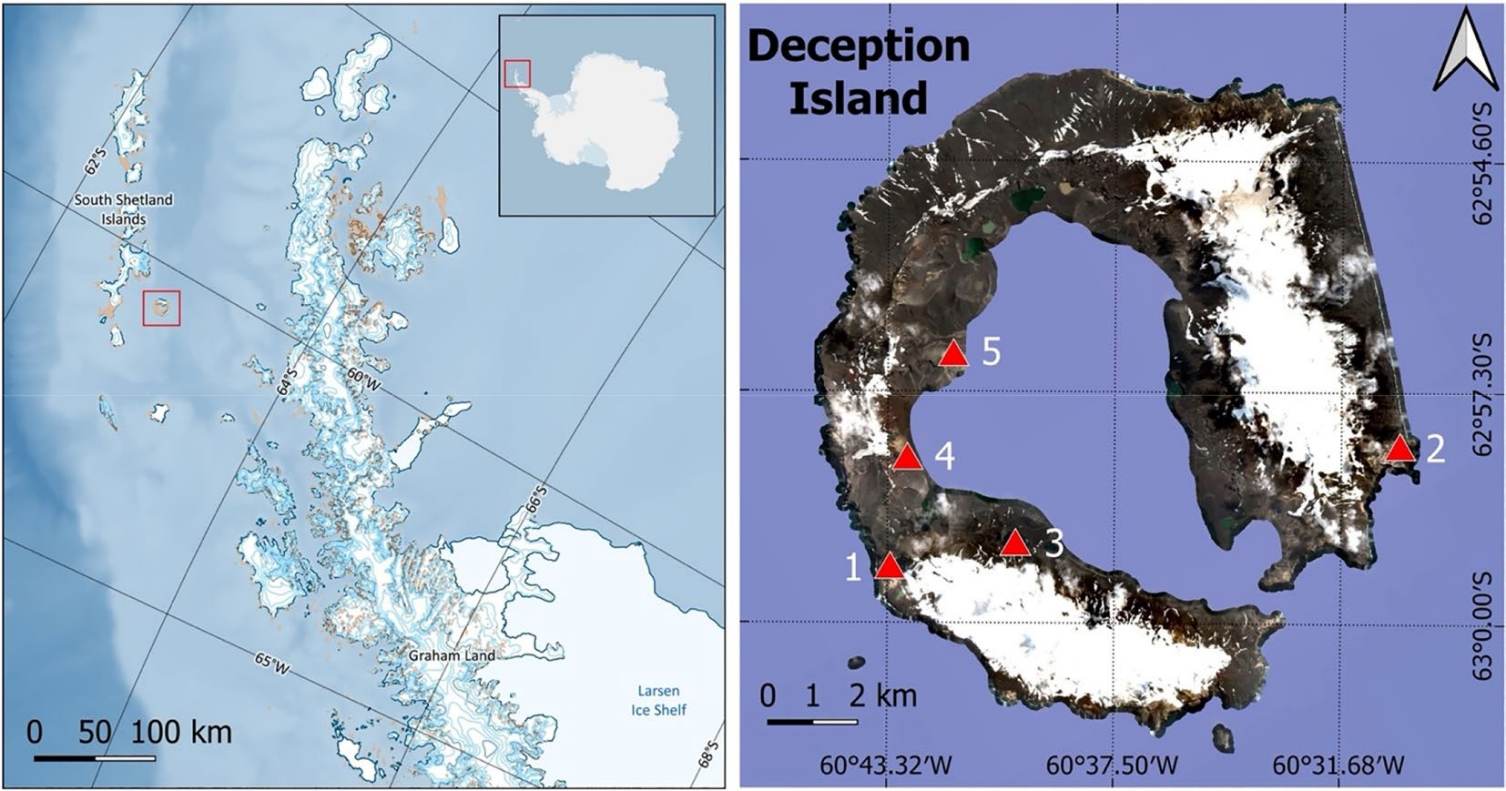
Location of Deception Island in the Antarctic peninsula and locations of UAV surveys carried out on Deception Island. 1: Vapour Col; 2: Baily Head; 3: Crater Lake; 4: Fumarole Bay; 5: Murature Formation. Data sources: Qantarctica package (https://www.npolar.no/quantarctica/) and Sentinel-2 L2A True Color image from January 19, 2020, respectively.

Different areas of Deception Island (62° 57′ S, 60° 38′ W) were surveyed in February 2021 in order to acquire visible, multispectral and/or thermal imagery using different sensors onboard UAVs, as well as surface water samples (Fig. 5; Table 1).

**Table 1:** Information and aims of UAV surveys carried out on Deception Island.

Location	UAV	Date (2021)	Camera/equipment	Ground sample distance (cm/pixel)	No. of captures	Flight time (min)	Target
Vapor Col Penguin Colony	Hexacopter	Feb 8th	Multispectral	6	3800	29	Thematic map
		Feb 8th	Thermal + RGB	8.5	336	29	Penguins identification
		Feb 18th	Water sampler	–	–	13	Physicochemical analysis
Murature formation	Quadcopter	Feb 9th	RGB	1.4	843	20	3D photogrammetry
Crater lake	Quadcopter	Feb 8–9th	Multispectral	6.5	3096	14	RGB Mosaic
	Hexacopter	Feb 9th	Water sampler	–	–	15	Physicochemical analysis
Bailey head	Hexacopter	Feb 14th	Thermal	5.3	203	22	Fur seals identification
	Hexacopter		Water sampler	–	–	15	Physicochemical analysis
	Quadcopter		RGB	1.8	309	13	RGB Mosaic
Fumarole bay	Quadcopter	Feb 19th	RGB	1	57	14	RGB Mosaic
	Hexacopter		Thermal	5.3	336	27	Thermal anolamlies identification

Imaging overlap selected was 80% frontal and 70% side.

### UAV platforms, sensors and equipments

Three UAVs were used in this study.

1. Hexacopter with three-bladed propellers with electric motor (130 kV brushless type) for each of the six blades (Condor, Dronetools). The maximum takeoff weight (MTOW) is 14.9 kg, and has a flight autonomy of 60 min (without payload). This UAV could be equipped with: (i) a MicaSense RedEdge-MX dual multispectral camera with 10 different spectral bands with the following wavelengths: coastal blue 444 nm, blue 475 nm, green 531 and 560 nm, red 650 and 668 nm, red edge 705, 717 and 740 nm, and near infrared (NIR) 842 nm (similar to Sentinel-2 satellite). The resolution of the sensor is 1280 × 960 pixels (8 cm/pixel from a height of 120 m) and has a horizontal field of view of 47.2°. It also has a Downwelling Light Sensor (DLS) with built-in GPS. The DLS provides more accurate and reliable measurements of irradiance and solar angle, improving radiometric accuracy and reducing post-processing time. A calibration panel (RP04-1924106-0B, MicaSense) was used for radiometric calibration; (ii) a radiometric thermal camera (FLIR Vue Pro R 19 mm) that captures non-contact temperature measurements with calibrated temperature data embedded in every pixel. The spectral band is 7.5–13.5 µm and has a sensor resolution of 336 × 256; (iii) a surface water sampling device. The system, hung from the drone on a 5-m rope, is composed of a peristaltic pump (working with a rechargeable Lipo battery, 12 V) equipped with acid-washed silicone tubing of 5 mm internal diameter attached to a PTFE cartridge filter (0.22 μm; Sartobran^®^). The system works at a constant flow rate of 150 mL/min and can collect up to 2 L of water in an acid clean LDPE bottle.
2. A quadcopter with an integrated RGB camera and a multispectral camera array with five bands covering Blue (450 nm), Green (560 nm), Red (650 nm), Red Edge (730 nm) Edge, and NIR (840 nm) (P4 Multispectral, DJI). The UAV includes an integrated spectral sunlight sensor to capture solar irradiance in order to maximize the consistency of data collection under different solar radiation. With a takeoff weight of 1487 g, it has a 27-min maximum flight duration.
3. A quadcopter with an integrated 24–48 mm Optical Zoom Camera (Mavic 2 zoom, DJI). With a takeoff weight of 905 g, it has a 31-min maximum flight duration.

### UAVs operational procedure

UAVs rules operations followed the Spanish civil aviation regulations whose responsible is the Spanish Agency for Aviation Safety (AESA). Accordingly, UAVs were operated by scientists with official UAVs Pilot licenses and the height flight limit was 120 m. Flights were planned with the Ground Station Pro (GS Pro) software (DJI), which considers geographical factors such as the elevation of the terrain, meteorological factors such as wind intensity or rainfall, and logistics such as the location of the area of interest for research. A series of constant parameters were previously established, such as the flight height (always above 50 m for ensuring the minimal disturbance on the fauna and up to 120 m for agreeing with the Spanish legislation), the flight speed (below 10 m/s to guarantee the quality data collected), the flight time (less than 30 min due to the autonomy of the batteries), ground sampling distance (GSD) (flight height dependent), the trajectory of the drone (parallel to main path), and the central and lateral overlaps (80% and 70%, respectively). For the multispectral sensor, before and after the flight, a calibration was made using a referenced reflectance panel (RP04-1924106-0B, MicaSense).

In order to guarantee minimal impact on the wildlife we followed the recommendations published by Hodgson and Koh^30^ and later by SCARS^31^. At the Chinstrap penguins’ colonies, best operational places were selected by moving away from the colony as much as necessary to ensure that the take-off and landing of the selected UAV did not cause any disturbance to the penguins.

### Software

For the generation of the photomosaic from the images obtained from the UAVs we used the software Pix4D Mapper (Pix4D SA, Lausanne, Switzerland). The WGS84 (EPSG: 4326) coordinate system was used to georeference the images. Once the images were imported into Pix4D Mapper, initial processing steps were performed, including the conversion of digital numbers into relative temperature (°C) for thermal imagery, the generation of the point cloud, the textured 3D mesh, the digital surface model (DSM), the orthomosaic, and the reflectance data for each spectral band. The radiometric processing and the calibration of the reflectance values of each band were performed from the calibration panel, which was photographed moments before the start of the flight, and with the DLS.

## Supplementary Information

Supplementary Figure S1.

Supplementary Video S1.
