# Supplementary material for: Applications of unmanned aerial vehicles in Antarctic environmental research
Source: Sci Rep. 2021 Nov 5;11:21717. doi: 10.1038/s41598-021-01228-z (PMC8571321; doi:10.1038/s41598-021-01228-z)
Supplement: Supplementary file 1 — Supplementary Figure S1. [file 41598_2021_1228_MOESM1_ESM.docx]

**Supplementary Information Appendix**

**Figure 1.** Lagoon in the penguin colony of Vapour Col with a bloom of red-pigmented green algae (*Chlorophyceae)*


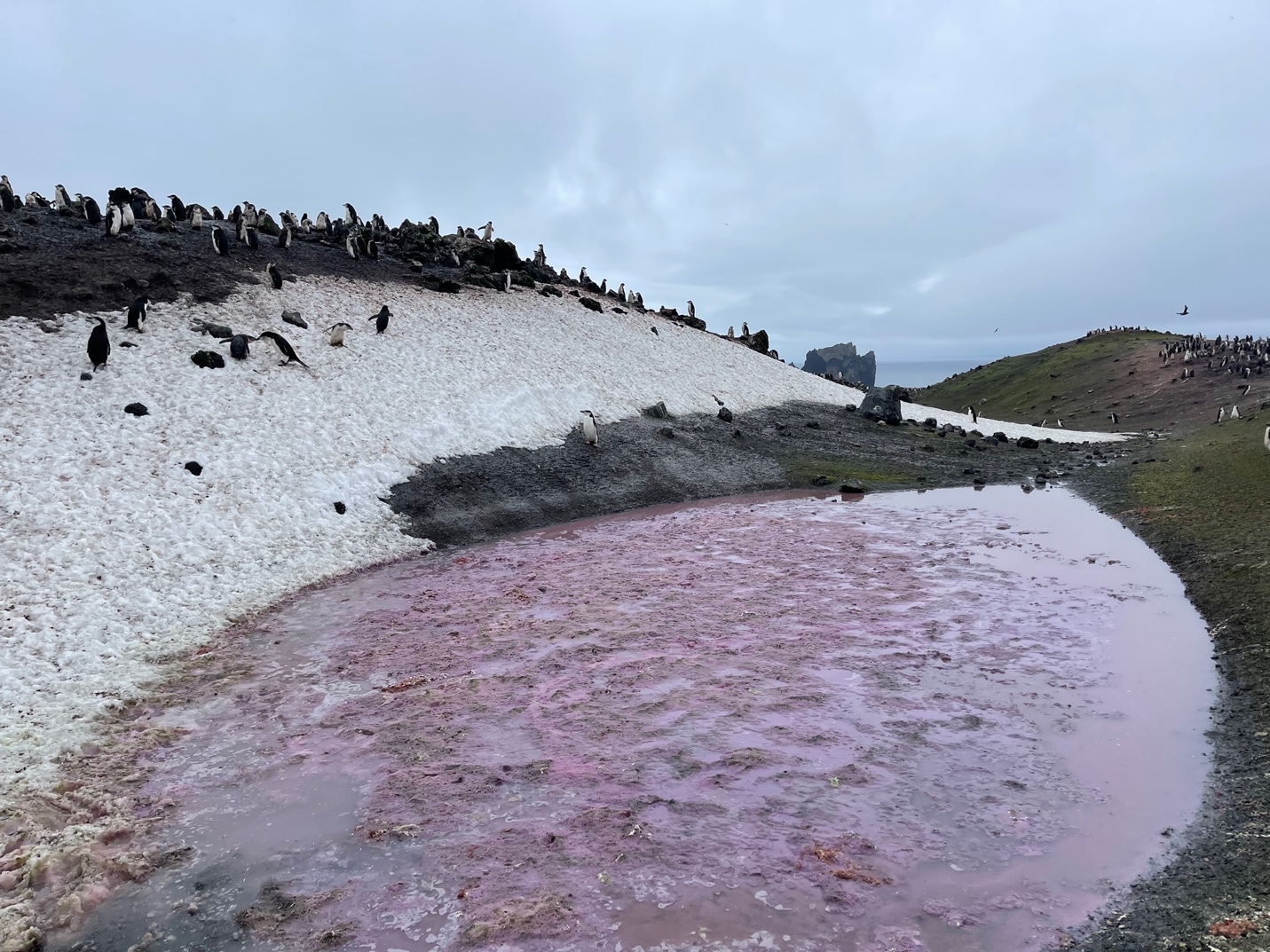


**Movie 1.** 3D Murature formation movie generated with 843 RGB pictures taken at 40 meters altitude from the quadcopter with integrated 24-48 mm Optical Zoom Camera.
